# Structural Elucidation of the DFG-Asp in and DFG-Asp out States of TAM Kinases and Insight into the Selectivity of Their Inhibitors

**DOI:** 10.3390/molecules191016223

**Published:** 2014-10-10

**Authors:** Abdellah Messoussi, Lucile Peyronnet, Clémence Feneyrolles, Gwénaël Chevé, Khalid Bougrin, Aziz Yasri

**Affiliations:** 1 OriBase Pharma, Parc Euromedecine, Cap Gamma, 1682, rue de la Valsière, 34189 Montpellier, France; E-Mails: amessoussi@oribase-pharma.com (A.M.); lpeyronnet@oribase-pharma.com (L.P.); cfeneyrolles@oribase-pharma.com (C.F.); gcheve@oribase-pharma.com (G.C.); 2 Laboratoire de Chimie des Plantes et de Synthèse Organique et Bioorganique, URAC23, Université Mohammed V, Faculté des Sciences B.P., 1014 Rabat, Morocco; E-Mail: khalid.bougrin@smct-ma.com

**Keywords:** tyrosine kinase, TAM kinase family, homology model, kinase selectivity

## Abstract

Structural elucidation of the active (DFG-Asp in) and inactive (DFG-Asp out) states of the TAM family of receptor tyrosine kinases is required for future development of TAM inhibitors as drugs. Herein we report a computational study on each of the three TAM members Tyro-3, Axl and Mer. DFG-Asp in and DFG-Asp out homology models of each one were built based on the X-ray structure of c-Met kinase, an enzyme with a closely related sequence. Structural validation and *in silico* screening enabled identification of critical amino acids for ligand binding within the active site of each DFG-Asp in and DFG-Asp out model. The position and nature of amino acids that differ among Tyro-3, Axl and Mer, and the potential role of these residues in the design of selective TAM ligands, are discussed.

## 1. Introduction

### 1.1. Kinases as Therapeutic Targets

Kinases are ubiquitous enzymes from the transferase family. Protein kinases catalyze the covalent transfer of a phosphoryl group from ATP to a substrate, which can be another protein (e.g., another kinase), a lipid or a nucleic acid. Protein kinases are the largest family of kinases, encompassing 518 members (as currently identified in the human genome) [1]. Their catalytic domain is well conserved and features a common set of crucial amino acids. All protein kinases possess an Mg^2+^-ATP binding site, a protein-substrate binding site and multiple regulator sites. One interesting class of protein kinases are receptor tyrosine kinases (RTKs), transmembrane proteins that transmit signals from the extracellular medium to the cytoplasm and nucleus. They are fundamental for basic life functions, as they regulate many cellular processes such as survival, growth, differentiation, adhesion and motility. Dysfunction or deregulation of certain kinases can lead to diseases such as cancer; thus, these enzymes have garnered enormous interest as therapeutic targets.

### 1.2. The TAM Family as Therapeutic Targets

The TAM family of protein RTKs was named after its three members: Tyro-3 (also called Sky), Axl (Ark or Ufo), and Mer (MerTK). TAM kinases contain three structural domains: an *extracellular domain*, which binds the natural ligand and comprises two immunoglobulin-like and two fibronectin domains [2]; an *intracellular* *domain*, which contains the tyrosine kinase domain and exhibits a well conserved sequence that defines the TAM family (KW-(I/L)-A-(I/L)-ES) [3]; and a *transmembrane domain*, which links the other two domains.

TAM kinases were initially found in cancer cells [4,5,6,7], which lead to the discovery that overexpression or ectopia of these RTKs contribute to carcinogenesis [8,9,10]. Indeed, many cancers, including cancers of the uterine endometrium [8], stomach [11,12], colon [13], prostate [14,15,16,17], thyroid [18,19,20], lung [21,22], breast [23,24,25,26], ovaries [27,28], liver [29] and kidneys [9], as well as glioblastomas [30], melanomas [31,32,33], osteosarcomas [34], leukemias [4,35,36] and multiple myelomas [37] exhibit altered expression of one or more TAM members [3]. Interestingly, among the three TAM members, Axl has been found to be the most involved in human cancers [3]. Moreover, in cancer Axl is implicated in drug-resistance mechanisms [38,39,40,41] and associated with poor prognosis and a high level of recurrence [42]. All of these findings corroborate the idea that TAM kinases are attractive potential targets for oncology treatment.

### 1.3. Structure-Based Drug Design for Lead Identification and Optimization

Over the past decade, identification of new drug targets has been greatly facilitated by the combination of classical biochemistry tools with new technologies such as proteomics. Furthermore, advances in analytical techniques such as NMR (higher frequency and technology) and crystallography (HTS, synchrotron radiation, phase resolution, *etc.*) have radically shortened the time needed to solve the structure of potential therapeutic targets. This progress has renewed interest in rational medicinal chemistry through Structure-Based Drug Design (SBDD), an approach that has gradually brought new drugs to market, including Viracept^®^ (nelfinavir; Agouron*)* [43,44] and Agenerase^®^ (amprenavir; Vertex and GlaxoSmithKline) [45]. 

### 1.4. Comprehension of Binding-Mode for the Design of Specific Enzyme Inhibitors

Understanding binding modes is especially important for kinases, given the high conservation of their catalytic domain, to which classical ATP competitive inhibitors (type I and II) tend to bind. Selectivity stems from interactions of the inhibitor with less conserved parts of the kinase domain. However, designing a synthetic inhibitor that can reach these *selectivity pockets* is not trivial: in the absence of a 3D structure of the target, ligand design requires intense Structure-Activity-Relationship (SAR) analyses and exhaustive chemical synthesis. 

The first step in SBDD studies is structural elucidation of the target, which can be done by X-ray crystallography or NMR. The next step is to assess the binding behavior between protein and ligand. If no data are available in the Protein Data Bank (PDB), then homology modeling can be used to this end.

Presently, there are no potent selective inhibitors of any TAM kinase on the market [46]. Given the widespread expression of these enzymes (Tyro-3 is found mainly in the central nervous system; Axl is ubiquitous; and Mer is found chiefly in macrophages and NK cells [3]), inhibitors of any single TAM must be highly selective. For kinase inhibitor drugs, selectivity is not merely a question of efficacy, it is also a requisite for safety. However, the lack of selectivity is tolerated in some therapeutical indications such as cancer.

In the work described here, we sought to study the activity domain of each of the three TAM kinases (Tyro-3, Axl and Mer) in each of their two conformations (DFG-Asp in and DFG-Asp out). Hence, we designed and validated relevant homology models, and then studied their active sites. We performed virtual screening of TAM inhibitors against these models, gaining insight into inhibitor/kinase selectivity and invaluable knowledge for the future design of scaffolds for new, active and selective TAM inhibitors.

## 2. Results and Discussion

### 2.1. Homology Modeling of the TAM Family

None of the TAM kinase 3D structures was solved in the DFG-Asp out conformation; thus, they were built by homology modeling using as template the phylogenetically-related tyrosine kinase c-Met in this conformation (PDB ID: 3F82 [47]). In fact, the identity percentages between each of the three TAM kinases and c-Met are above 45%: the values are 45.42% for Tyro-3, 45.98% for Axl and 45.04% for Mer.

Crystal structures of Mer and Tyro-3 in the DFG-Asp in conformation were published in 2009 (PDB ID: 2P0C, 3BRB, 3BPR [48]), 2012 (PDB ID: 3TCP, 3QUP) [49,50] and 2013 (PDB ID: 4M3Q, 4MH7, 4MHA, 4FEQ, 4FF8 [51]). However, all these 3D structures correspond to murine proteins in their DFG-Asp in state and lack a portion of the activation loop. Consequently, as we wanted to study the whole kinase domain with its activation loop for the human kinases, we decided to build 3D models for the three kinases in the DGF-Asp in state using X-ray structure of c-Met kinase as a template. This 3D structure corresponds to the human c-Met kinase and it has the activation loop completely characterized (PDB ID: 2WD1 [52])).

### 2.2. Validation of the TAM Kinase Models

**Figure 1:**
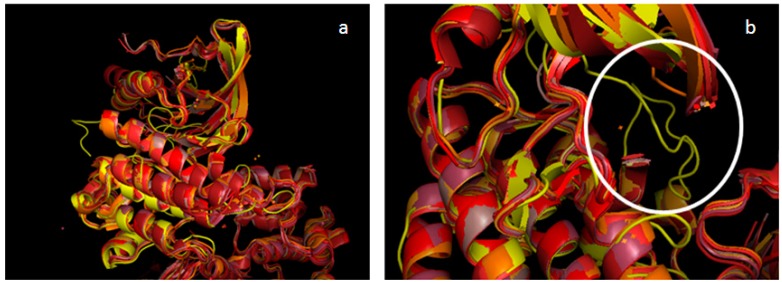
Alignment of all Mer structures from PDB with our Mer DFG-Asp in model. PDB structure of Mer in different shades of red (PDB ID 2P0C in *dark salmon*; PDB ID 3BPR in *TV red*; PDB ID 3BRB in *firebrick*; PDB ID 3TCP in *raspberry*; PDB ID 4M3Q in *orange*, PDB ID 4MH7 in *bright orange*; PDB ID 4MHA in *red*); our Mer DFG-Asp in model, in *yellow* (all colors from PyMOL). (**a**) Superimposition of reported Mer crystal structures with our Mer DFG-Asp in model. (**b**) Zoom on the activation loop (circled in white) found only in our model.

**Figure 2:**
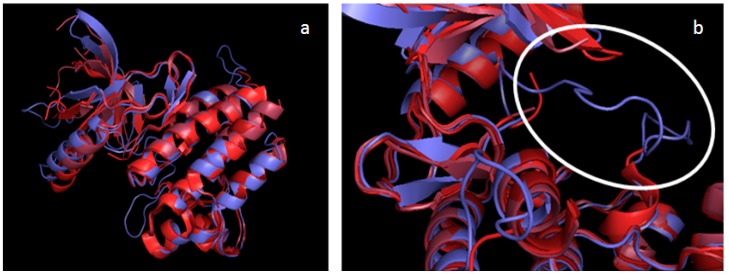
Alignment of all Tyro-3 structures from PDB with our Tyro-3 DFG-Asp in model. PDB structure of Tyro-3 in different shades of red (PDB ID 3QUP in TV red; PDB ID 4FF8 in firebrick; PDB ID 4FEQ in raspberry); our Tyro-3 DFG-Asp in model, in slate blue (all colors from PyMOL). (**a**) Superimposition of Tyro-3 crystal structures with our Tyro-3 DFG-Asp in model. (**b**) Zoom on the activation loop (circled in white) found only in our model.

A total of six models was built (three TAM kinases x two states), and then structurally validated by checking the torsion angles for each amino acid. These calculations were performed using Procheck software, which generates Ramachandran plots. The three DFG-Asp out models possess 88.4% (Tyro-3), 87.9% (Axl) and 86.8% (Mer) of the amino acids in the favorable regions; and the three DFG-Asp in models, 90.8% (Tyro-3), 88.8% (Axl) and 87.7% (Mer) (Supplementary Figure S1). The amino acids outside of the favorable region are located on the protein surface, which is exposed to the solvent and is not subjected to the docking process. Since the DFG-Asp in crystal structures of Mer and of Tyro-3 in the literature are incomplete, we further validated our DFG-Asp in models of these two TAM kinases by superimposing them over the corresponding reported structures (Figure 1 and Figure 2).

We also validated each of our six models, by docking a known efficient inhibitor into each one. For the DFG-Asp out models, the docking ligand used was BMS-777607, as it has previously been crystallized with the template kinase, c-Met (IC_50_ = 3.9 nM), and is active against all three of the TAM kinases (from highest to lowest activity: IC_50_ Axl = 1.1 nM; IC_50_ Tyro-3 = 4.3 nM; and IC_50_ Mer = 14 nM).

**Figure 3:**
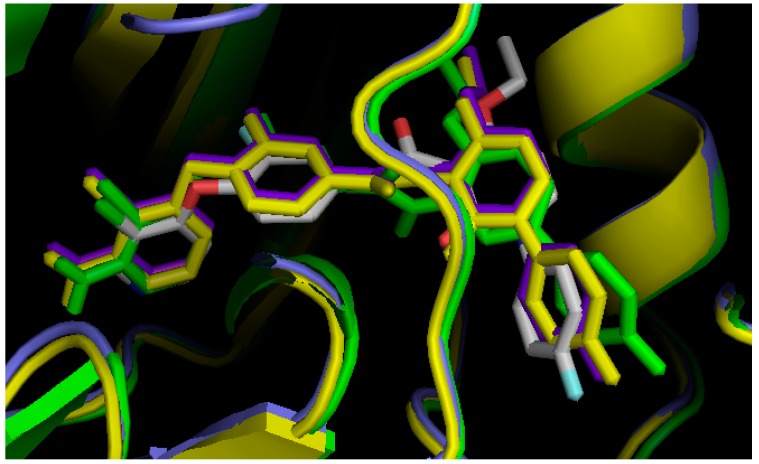
*In silico* screening of the DFG-Asp out homology models, using BMS777607. Tyro-3 in *purple*; Axl in green; Mer in *yellow*; and BMS777607 from the crystal structure of c-Met (PDB ID:3F82), in white. In each case, the color of BMS777607 is the same as that of the docking target.

The position of BMS-777607 in the active site of three TAM kinases in the DFG-Asp out state is very closely related to its crystallized position in c-Met (PDB ID: 3F82) (Figure 3). Thus, the three DFG-Asp out models were validate again by a different method.

**Figure 4:**
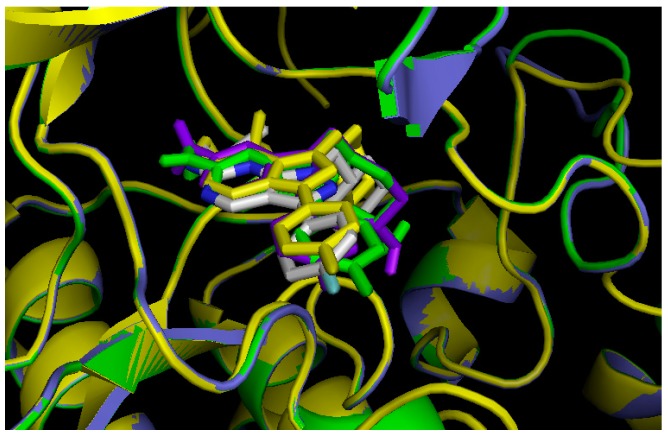
*In silico* screening of the DFG-Asp in homology models, using UNC569. Tyro-3 in purple, Axl in green; Mer in yellow; and UNC569 from the crystal structure of Mer (PDB ID: 3TCP), in white. In each case, the color of UNC569 is the same as that of the docking target.

For the DFG-Asp in models of Tyro-3 and of Axl, the docking ligand used was UNC569, as it has already been crystallized with Mer (PDB ID: 3TCP; IC_50_= 2.9 nM) and inhibits both of these TAM kinases (IC_50_ Tyro-3 = 48 nM; and IC_50_ Axl = 37 nM) [53]. The position of UNC569 in each of the three TAM kinases is close to that in the crystal structure of Mer (Figure 4). Hence, the three DFG-Asp in models were validated another time.

### 2.3. Comparison of Active Sites

To account for the variability in the active sites among the three TAM kinases in each conformation, we delimited volumes at 4 Å, 5 Å and 6 Å from any atom of the docked ligands, using PyMol software. 

**Figure 5:**
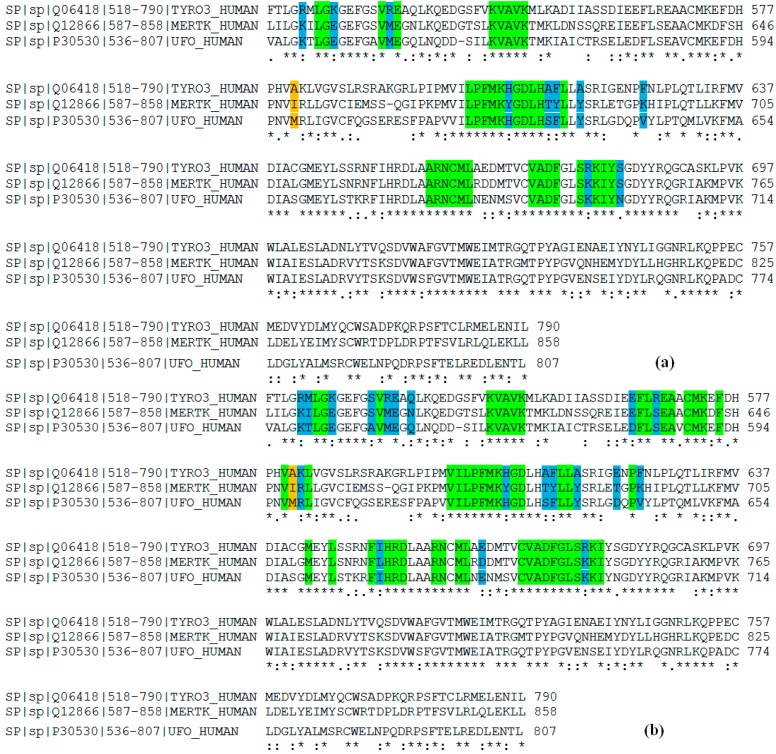
Alignment of the three TAM kinase domains. The active site is highlighted at 6 Å from the docked ligands. Identical residues are shown in green; non-conserved residues; in blue; and the key residue for each kinase, in orange. (**a**) Sequence alignment for the DFG-Asp in conformation. (**b**) Sequence alignment for the DFG-Asp out conformation.

The ligands used for delimiting these volumes were BMS-777607 (crystallized with C-Met in its DFG-Asp out state; PDB ID: 3F82) for the DFG-Asp out state, and UNC569 (crystallized with Mer in its DFG-Asp in state; PDB ID: 3TCP) for the DFG-Asp in state. We then enumerated all the amino acid sequences contained within these volumes. As representative examples, we show here the set of amino acids encompassed at 6 Å for each TAM kinase model in the DFG-Asp in (Figure 5a) and the DFG-Asp out (Figure 5b) conformations. 

The sequence alignments, and the active sites defined at distances of 4, 5 or 6 Å from the ligands, revealed a conserved sequence formed comprising one Leu residue (serving as gatekeeper) and the triplet Pro-Phe-Met (forming a hinge between the N-terminal lobe and the C-terminal lobe). The gatekeeper Leu regulates access to the hydrophobic specificity pocket, which comprises an Ala-Asp-Lys sequence followed by a single residue that differs with each TAM kinase (Ala in Tyro-3; Met in Axl; or Ile in Mer). 

At 4 Å, the active site encompasses 45 amino acids, seven of which differ among the three TAM kinases. At 5 Å, the active site spans 59 amino acids, fifteen of which differ among the three kinases. Finally, at 6 Å, the active site is composed of 73 amino acids, nineteen of which differ by the TAM kinase. Analysis of the 3D-homology models of the three kinases revealed that, based on the orientations in the active site, there is only one *key residue* in both states, whose side chain is oriented inside the active site: Ala581 in Tyro-3; Met598 in Axl; and Ile650 in Mer (Figure 5). We reasoned that this amino acid must play a crucial role in the selectivity of ligands for each of the three TAM kinases. The 3D analysis also revealed that for all the active sites in each of the two conformations (DFG-Asp in and DFG-Asp out), the activation loop of the kinase domain formed part of the active site. This finding underscores the importance of using a model that includes the residues of the activation loop.

### 2.4. Virtual Screening

We sought to investigate the selectivity of ligands among the three TAM kinases, as well as the role of the key residue in each kinase (identified in the previous section), using virtual screening of known TAM inhibitors. Thus, we searched two databases (PubChem and CanSAR) and obtained a set of 156 compounds (Supplementary Table S1). Depending on the chemical structure, these ligands were docked into each member of the TAM family in either the DFG-Asp in (151 compounds) or the DFG-Asp out (five compounds) conformation. 

Given the high number of ligands docked in the DFG-Asp in conformation models, we required a data-sorting step. We performed this step using a designed scoring function in an MS Excel (MS Office 2010) spreadsheet, which enabled us to ascertain the inhibition profile of each compound based on its IC_50_ values (Supplementary Table S1). This procedure afforded 29 ligands with a strong inhibition profile: five that are selective against Tyro-3; 22 that are selective against Mer; and two pan-inhibitors of the TAM kinases. By analyzing the docking results for these 29 compounds, and taking into consideration their IC_50_ values, we were able to determine the role of the key residue of each TAM kinase in ligand selectivity. The selected inhibitors were then divided into two chemical series based on their common structural cores: diaminopyrimidines and aminopyrazolopyrimidines.

### 2.5. Insight into Selectivity

#### 2.5.1. Diaminopyrimidine Scaffold

**Figure 6:**
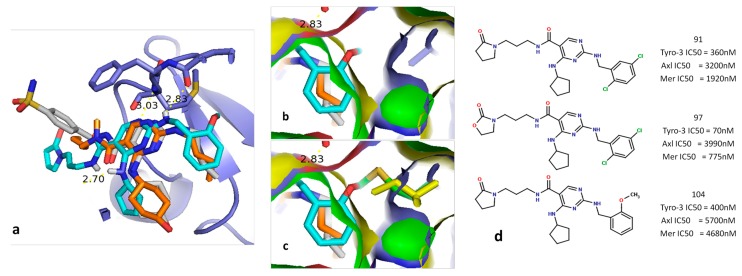
Comparison and overlapping of compound **104** docked in the Tyro-3 model aligned with the PDB ligands UNC1817 (PDB: 4MHA) and UNC1896 (PDB: 4MH7). (**a**) Overview of the overlapping of the three ligands in the active site of the Tyro-3 model. (**b**) Overview of the distance between the methoxyphenyl fragment in compound **104** and the Ala581 side chain of Tyro-3. (**c**) Overview of the distance between the methoxyphenyl fragment in compound **104** and either Met598 in Axl or the Ile650 side-chain in Mer. (**d**) Representation of the three diaminopyrimidine-based inhibitors selective for Tyro-3.

For the first series, three inhibitors selective for Tyro-3 were docked into the Tyro-3 homology model. Analysis of the docking results revealed that all three inhibitors adopt the same position in the Tyro-3 active site as that seen in the crystallized structures of Mer containing inhibitors bearing the same structural core (PDB ID: 4MHA and 4MH7) [51]. For instance, with compound **104**, three hydrogen bonds formed between the diaminopyrimidine moiety of the ligand and the backbones of three residues from the active site of the Tyro-3 model: the two hinge residues (Pro604 and Met606), and one residue in the roof of the ATP-binding site (Leu524) (Figure 6).

The study of the aforementioned docking conformation revealed that, as previously supposed by Zhang *et al.* [51], the overlapping key residues from each TAM kinase (Ala581 in Tyro-3; Met598 in Axl; and Ile650 in Mer) help determine the selectivity of a given ligand. As clearly shown in Figure 6, the methoxyphenyl group of compound **104** comes over Ile650 in Mer and Met598 in Axl (the methoxy fragment passes through the surface of Mer (in yellow) and of Axl (in green)), whereas it has much more space in Tyro-3, due to the small size of the Ala581 side chain. Similar steric constraints are observed for the 5-chloride group in compounds **91** and **97** when either ligand is docked inside the Tyro-3 model and subsequently overlapped with the Axl and Mer homology models. Hence, we reasoned that the key variable residue could be exploited in the design of selective, type I Tyro-3 inhibitors.

#### 2.5.2. Pyrazolopyrimidine Scaffold

**Figure 7:**
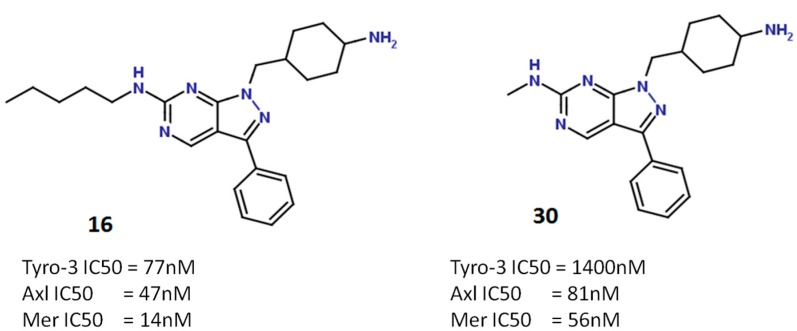
Structure of compounds **16** (**left**) and **30** (**right**), and their respective activities against Tyro-3, Axl and Mer.

Our second series of ligands comprised 36 compounds designed around a pyrazolopyrimidine scaffold. Docking of these molecules in each of our three DFG-Asp in models (Tyro-3, Axl and Mer) confirmed the binding mode discovered by Liu *et al.* [49]: formation of two hydrogen bonds between the pyrazolopyrimidine scaffold and the hinge region (Pro672 and Met674 in Mer), and of two hydrogen bonds between the 4-aminocyclohexyl moiety and residues Arg727 and Asn728. All the active compounds in this series established these same four hydrogen bonds with the conserved amino acids of the three TAM kinase active sites. Thus, selectivity amongst the kinases can be explained only by the previously described differential key residue. For example, in compound **30**, the methylamine substituent at position 6 of the scaffold (Figure 7) can establish hydrophobic contacts with Ile650 in Mer or with Met598 in Axl, giving this inhibitor strong activities against these two kinases (IC_50_ Mer = 56 nM; and IC_50_ Axl = 81 nM). 

**Figure 8:**
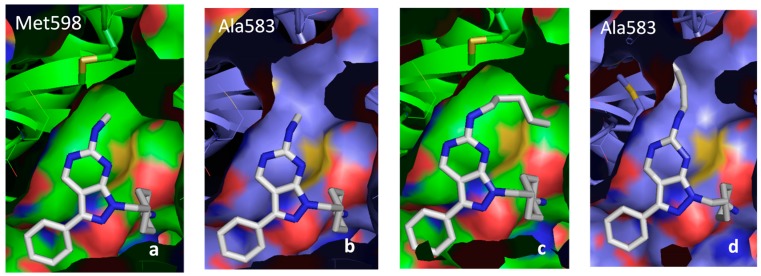
Docking of compounds **30** and **16** in the Axl and Tyro-3 models. Representation of the docking of compound **30** in the active site of the Axl model (**a**) and of the Tyro-3 model (**b**). Interaction of the pentylamine substituent of compound **16** with Met-598 in Axl (**c**), or with the hydrophobic pocket created by Ala-581, Ala-583, Pro-604, Met-606 and Ala-672 in the active site of the Tyro-3 model (**d**).

However, as the methylamine substituent is too short to interact with Ala583 in Tyro-3, the activity of compound **30** against this kinase is much weaker (IC_50_ Tyro-3 = 1400 nM) (Figure 8). In contrast, compound **16**, which features a pentylamine substituent, is very active against all three kinases (from highest to lowest activity: IC_50_ Mer = 14 nM; IC_50_ Axl = 47 nM; and IC_50_ Tyro-3 = 77 nM). Interestingly, the pentylamine substituent of compound **16** is sufficiently long to enter the hydrophobic pocket in Tyro-3, where it interacts with the side-chain of Ala583 as well as other surrounding hydrophobic amino acids (Ala581, Pro604, Met606 and Ala672). This difference in surface size explains why type I inhibitors such as compound **30** are more selective for Axl and Mer than for Tyro-3.

#### 2.5.3. DFG-Asp out Docking Analysis

**Figure 9:**
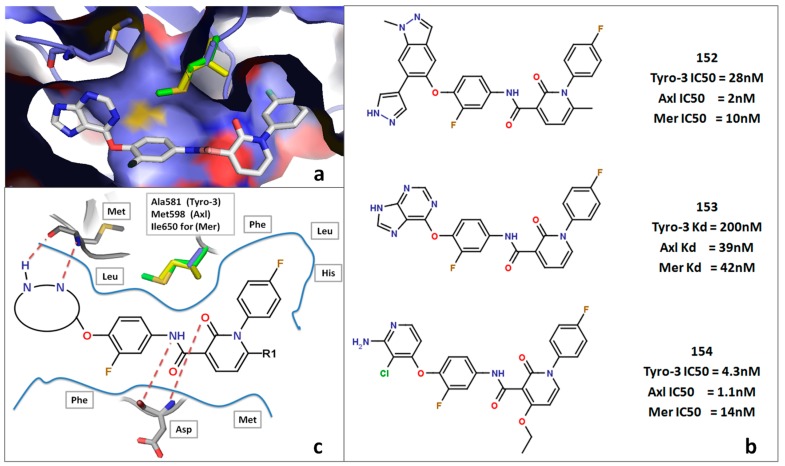
DFG-Asp out study. (**a**) Docking of compound **153** in the active site of the Mer DFG-Asp out model. (**b**) Structure of compounds **152**–**154** with their respective activities against Tyro-3, Axl and Mer. (**c**) General schematic of type II inhibitors docking in the active site of a TAM kinase in the DFG-Asp out conformation.

To analyze the DFG-Asp out conformations among the three TAM kinases, we collected five TAM DFG-Asp out inhibitors from online bioassay databases (Supplementary Table S2), and then docked each one in each of our DFG-Asp out models (Tyro-3, Axl and Mer). Upon close analysis of the docking of compounds **152**–**154** we identified the same orientation and binding mode in our models as those reported by Suarez *et al.* [54]. In this scenario, the purine core forms two hydrogen bonds with the hinge Met residue, the fluorophenyl group generates π-stacking interactions with the Phe of the DFG-Asp; the amide linker forms a hydrogen bond with the Asp of the DFG-Asp sequence, which also interacts with the carbonyl of the pyridine; and the terminal *para-*fluorophenyl moiety goes inside a hydrophobic pocket formed by Phe, Leu, and His in all three kinases (Figure 9). All of these interactions generate a potent type II TAM kinases inhibitor [54]. For the fluorine atom on the terminal phenyl moiety, the *para* position is that which leads to the highest activity; however, it generally corresponds to pan-TAM kinases inhibitors. Other highly potent TAM inhibitors such as compounds **152** and **154** bear the same general structure but differ in the heterocyclic group that binds to the hinge [46,47].

## 3. Experimental Section

### 3.1. Data Sources

There are 156 TAM inhibitors whose structures and IC_50_ values for each TAM enzyme are currently available; all of these data were retrieved from the CanSAR and PubChem databases [47,49,50,51,54,55,56,57,58,59,60,61,62,63]. The 3D structures of the template kinase c-Met in the DFG-Asp in and the DFG-Asp out conformations (2WD1 [52] and 3F82 [47], respectively) were retrieved from the Protein Data Bank [64]. The primary sequences of Tyro-3, Axl, Mer and c-Met were retrieved from the UniProt database (Uniprot ID: Q06418, P30530, Q12866, P08581 respectively) [65]. 

### 3.2. Homology Modeling

Sequences were aligned using CLUSTAL O (1.2.1) [66] multiple-sequence alignment from the Align tool of UniProt. The Swiss model server [67] was used to build the homology models of Tyro-3, Axl and Mer in each state (DFG-Asp in and DFG-Asp out). The templates used were the c-Met crystallized structures in either state. The obtained models were structurally validated using Procheck software [68]. Another validation was also performed using known inhibitors of TAM kinases. 

### 3.3. Docking

We ran all 156 of the retrieved TAM inhibitors through virtual screening. Of these, 151 compounds docked in the three TAM kinase DFG-Asp in conformations, whereas five compounds docked in the corresponding DFG-Asp out conformations. Energy minimization was performed using Swiss Pdb-Viewer (v4.1.0) [69]. 

The docking files (ligand + protein active site) were prepared with Autodock Vina software [70], by using AutoDock Tools (v1.5.6) [71]. The following parameters were adjusted in this preparation step: (1) the Gastieger charges and polar hydrogens were added; (2) the grid-box dimensions were set at 20 Å (X), 16 Å (Y) and 24 Å (Z) for the DFG-Asp in conformation, and 16 Å (X), 16 Å (Y) and 28 Å (Z) for the DFG-Asp out conformation; and (3) the center of the box was positioned at the midpoint of the active site, and the box volume covered the entire active site area plus a significant portion of the protein’s solvent-exposed surface. 

The following docking parameters were used in Autodock Vina: (1) all bonds in the inhibitor structures were allowed to rotate freely, except for the multiple bonds and the bonds in aromatic entities; (2) the kinase 3D structures were considered to be rigid; (3) a Lamarckian genetic algorithm was used for searching the conformational space in the active site; (4) the default grid spacing was set at 0.375 Å; (5) 100 different conformations were assessed, and the 20 highest scored binding modes were maintained for visual inspection; (6) the maximum energy difference between the best and the worst binding modes was set at 3 kcal/mol; (7) the scoring function was a stochastic global optimization method inspired chiefly by X-Score [72]; and (8) visual inspection of the docking results, and image building, were done using PyMOL software [73].

## 4. Conclusions

The TAM receptor tyrosine kinases (Tyro-3, Axl and Mer) have been widely implicated in the development of cancer and other diseases; thus, they have become therapeutic targets of interest. In this study, we built homology models of each TAM kinase in each of its two states (DFG-Asp in and DFG-Asp out). We validated these models by two different methods, and then successfully overlapped them with existing partial 3D structures of Tyro-3 and of Mer. We highlighted the active site for each conformation and identified a single key residue that varies among the three kinases. We found a large set of type I TAM inhibitors in the literature, docked them in our three DFG-Asp in models, and subsequently sorted them according to their inhibition profiles, which paralleled the aforementioned key variable residue. This provided knowledge that could be exploited for the future SBDD of TAM inhibitors selective for Tyro-3 or for Mer/Axl.

We also explored type II inhibitors, which bind to the TAM kinases in their DFG-Asp out conformations. As we found only a few inhibitors with defined activity against the TAM kinases, we did not have sufficient data with which to define a clear selectivity pattern. Moreover, we found that the key variable residue, which is paramount in the type I binding, is not very involved in the type II binding. However, we also determined that a terminal *para*-fluorophenyl group favors potent type II pan-TAM inhibitors. Further studies on additional type II inhibitors with defined TAM activities might enable a better understanding of the selectivity. We are presently using our findings and models from this study to design and synthesize new chemical entities as selective type I or type II TAM inhibitors.

## Supplementary Materials

Supplementary materials can be accessed at: http://www.mdpi.com/1420-3049/19/10/16223/s1.

## Supplementary Files

Supplementary File 1
