# Supplementary material for: Structural Elucidation of the DFG-Asp in and DFG-Asp out States of TAM Kinases and Insight into the Selectivity of Their Inhibitors
Source: Molecules. 2014 Oct 10;19(10):16223–39. doi: 10.3390/molecules191016223 (PMC6271404; doi:10.3390/molecules191016223)
Supplement: Supplementary File 1 [file molecules-19-16223-s001.pdf]

Supplementary Materials

Figure S1. Ramachandran plots of the six models built by homology modeling from c-Met template. The three first ones illustrate the structural validation of the DFG-in conformations of Tyro-3, Axl and Mer. The three last ones illustrate the structural validation of the DFG-out conformations of Tyro-3, Axl and Mer.

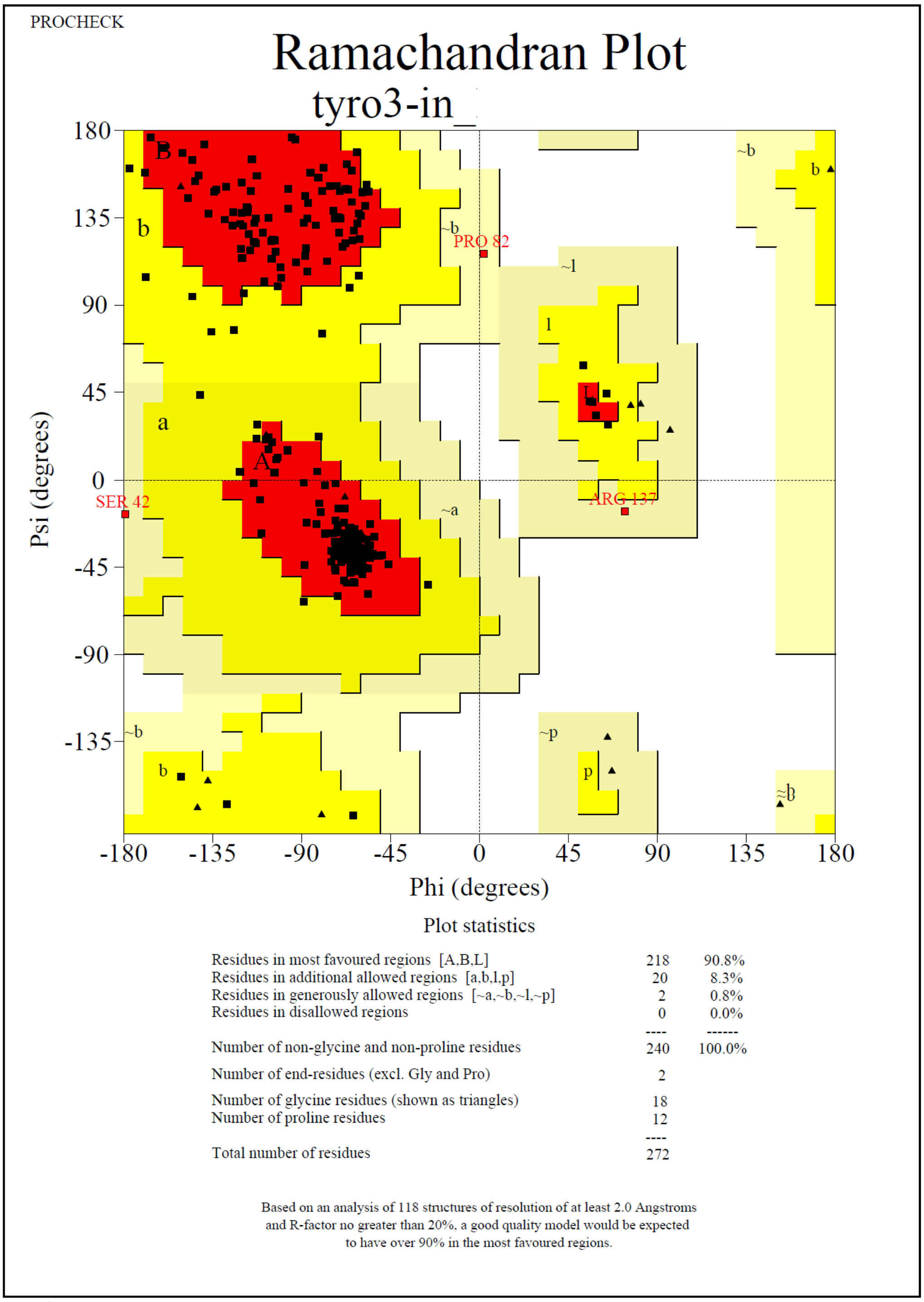

tyro3-in_

Figure S1. *Cont.*

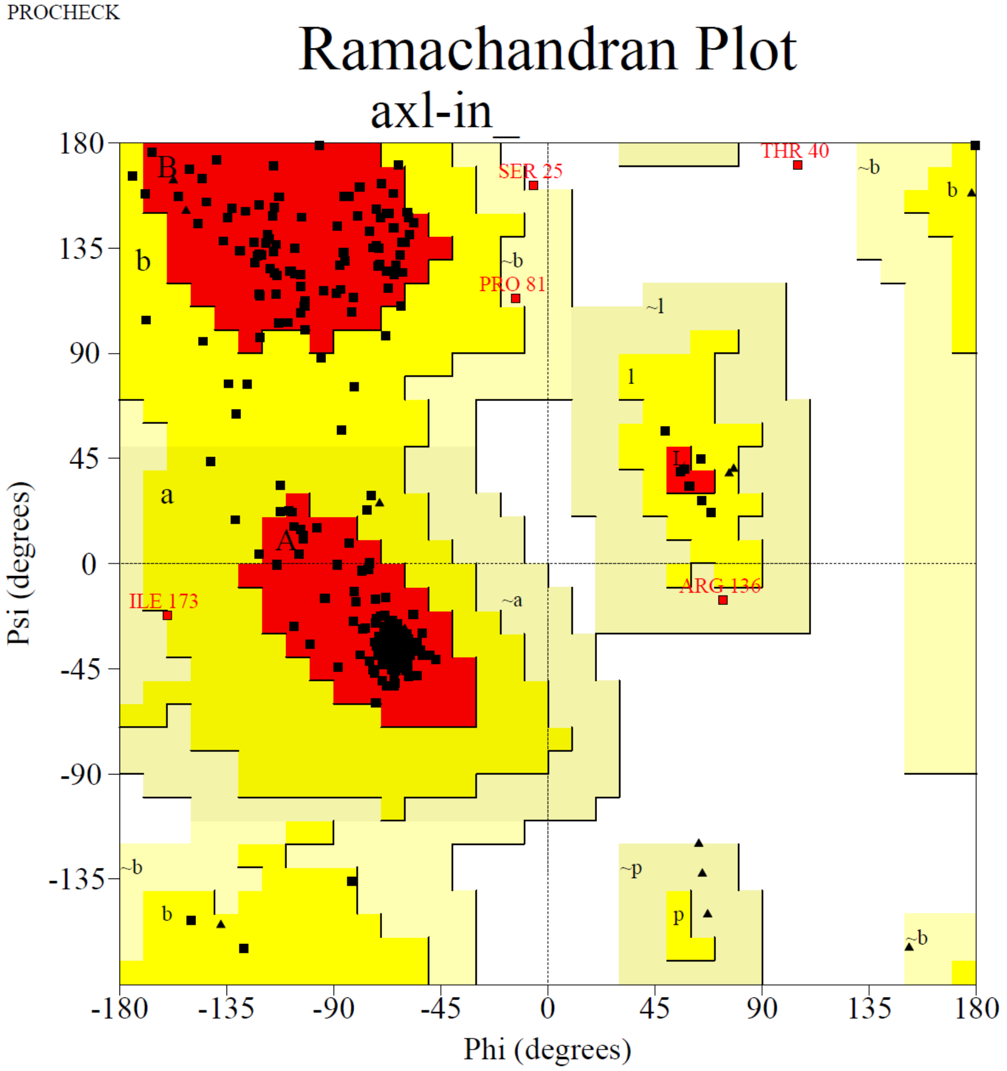

Plot statistics

Residues in most favoured regions [A,B,L]	213	88.8%
Residues in additional allowed regions [a,b,l,p]	23	9.6%
Residues in generously allowed regions [~a,~b,~l,~p]	2	0.8%
Residues in disallowed regions	2	0.8%
	----	------
Number of non-glycine and non-proline residues	240	100.0%
Number of end-residues (excl. Gly and Pro)	2	
Number of glycine residues (shown as triangles)	18	
Number of proline residues	12	

Total number of residues	272	

Based on an analysis of 118 structures of resolution of at least 2.0 Angstroms and R-factor no greater than 20%, a good quality model would be expected to have over 90% in the most favoured regions.

axl-in_

Figure S1. *Cont.*

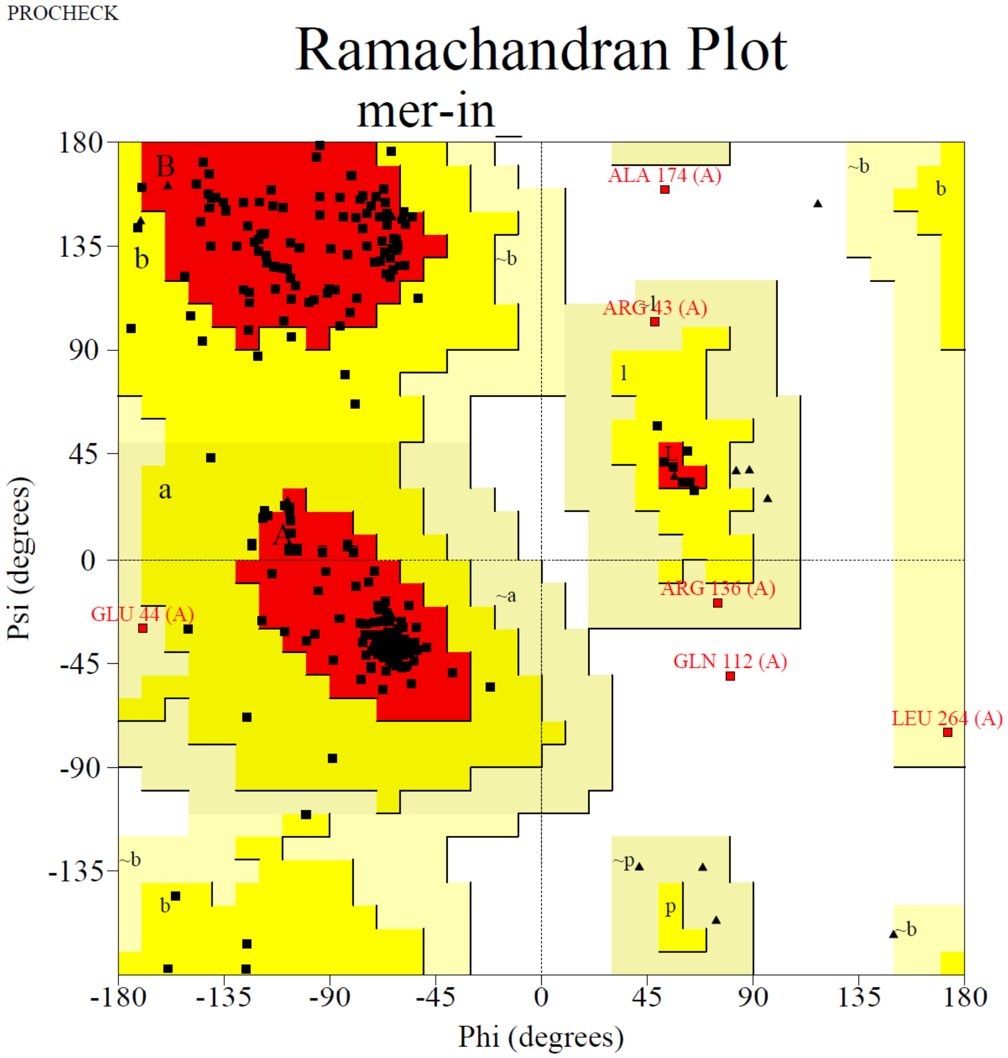

Plot statistics

Residues in most favoured regions [A,B,L]	206	87.7%
Residues in additional allowed regions [a,b,l,p]	23	9.8%
Residues in generously allowed regions [~a,~b,~l,~p]	4	1.7%
Residues in disallowed regions	2	0.9%
	----	------
Number of non-glycine and non-proline residues	235	100.0%
Number of end-residues (excl. Gly and Pro)	2	
Number of glycine residues (shown as triangles)	19	
Number of proline residues	12	

Total number of residues	268	

Based on an analysis of 118 structures of resolution of at least 2.0 Angstroms and R-factor no greater than 20%, a good quality model would be expected to have over 90% in the most favoured regions.

mer-in_

Figure S1. *Cont.*

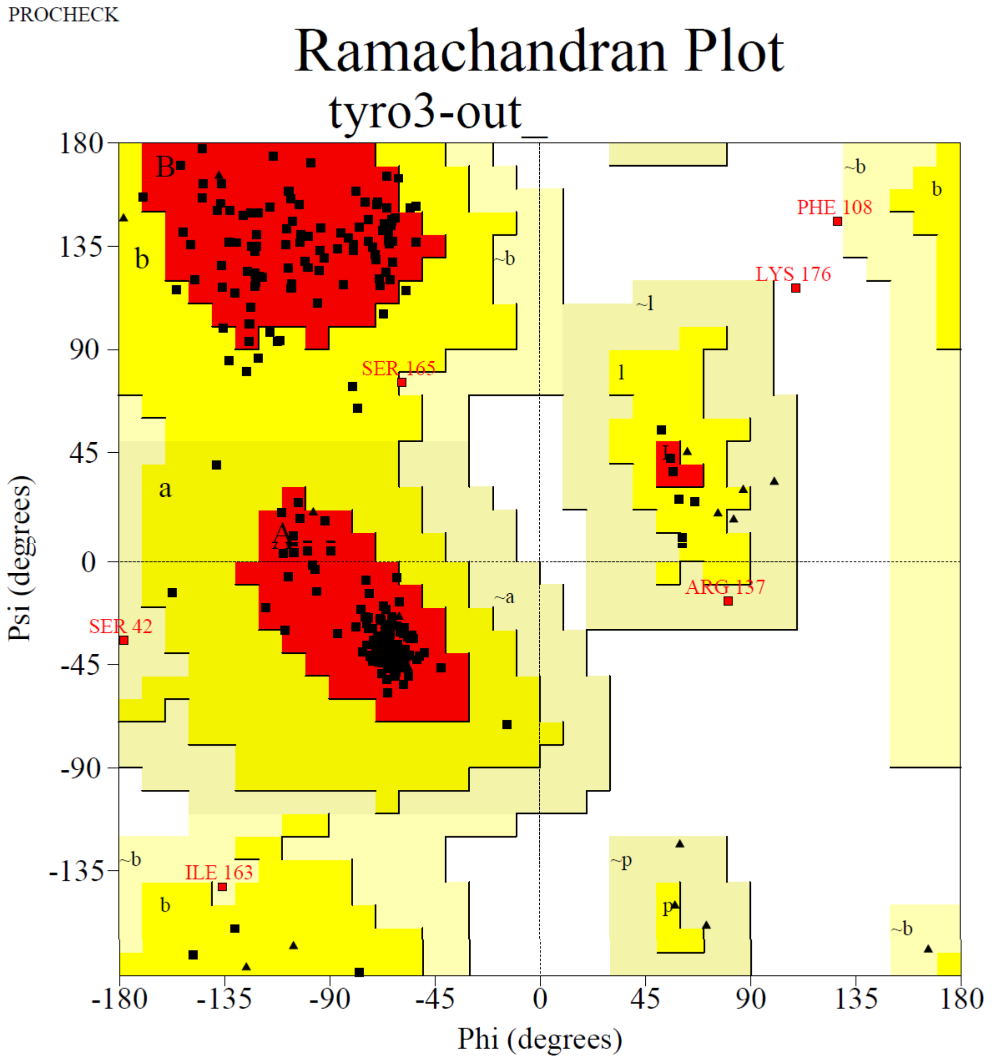

Plot statistics

Residues in most favoured regions [A,B,L]	213	88.4%
Residues in additional allowed regions [a,b,l,p]	22	9.1%
Residues in generously allowed regions [~a,~b,~l,~p]	4	1.7%
Residues in disallowed regions	2	0.8%
	----	------
Number of non-glycine and non-proline residues	241	100.0%
Number of end-residues (excl. Gly and Pro)	2	
Number of glycine residues (shown as triangles)	18	
Number of proline residues	12	

Total number of residues	273	

Based on an analysis of 118 structures of resolution of at least 2.0 Angstroms and R-factor no greater than 20%, a good quality model would be expected to have over 90% in the most favoured regions.

tyro3-out_

Figure S1. *Cont.*

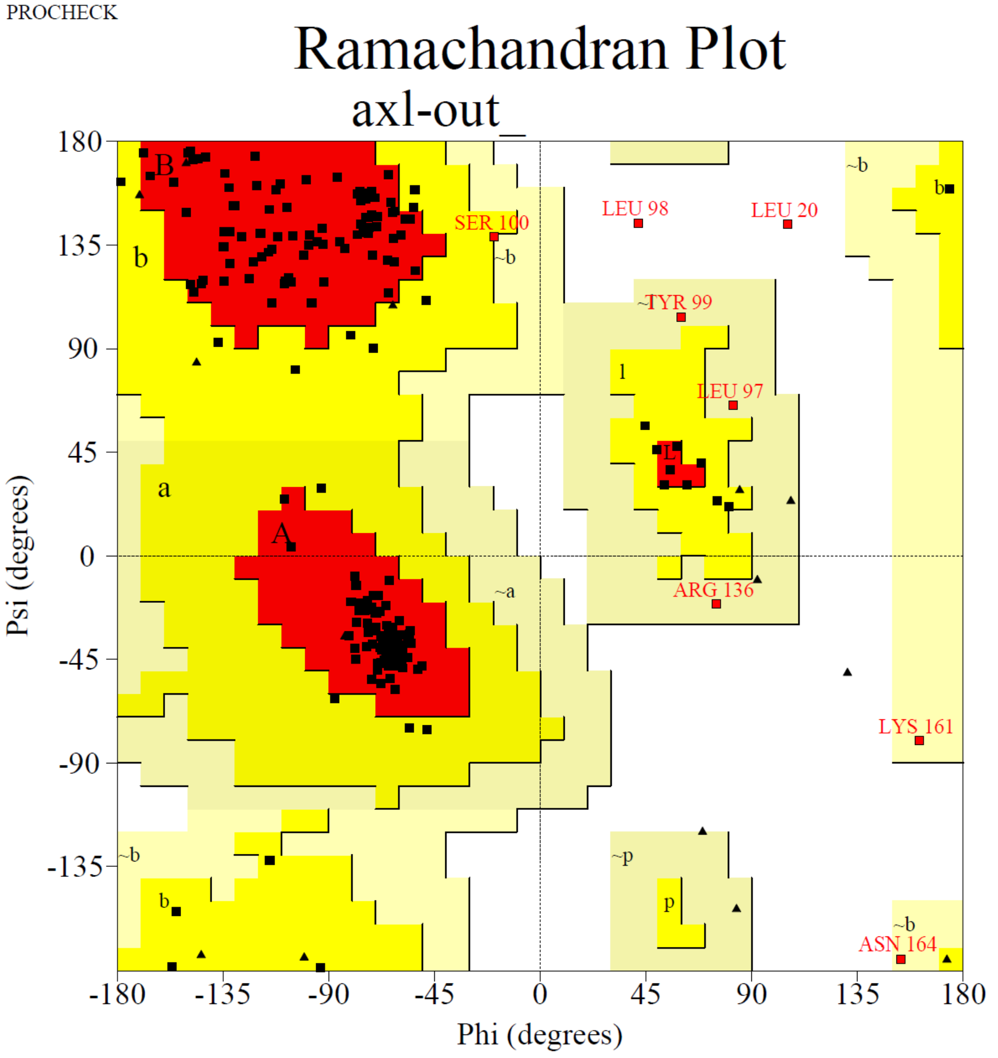

Plot statistics

Residues in most favoured regions [A,B,L]	204	87.9%
Residues in additional allowed regions [a,b,l,p]	20	8.6%
Residues in generously allowed regions [~a,~b,~l,~p]	6	2.6%
Residues in disallowed regions	2	0.9%
	----	------
Number of non-glycine and non-proline residues	232	100.0%
Number of end-residues (excl. Gly and Pro)	2	
Number of glycine residues (shown as triangles)	18	
Number of proline residues	12	

Total number of residues	264	

Based on an analysis of 118 structures of resolution of at least 2.0 Angstroms and R-factor no greater than 20%, a good quality model would be expected to have over 90% in the most favoured regions.

axl-out_

Figure S1. *Cont.*

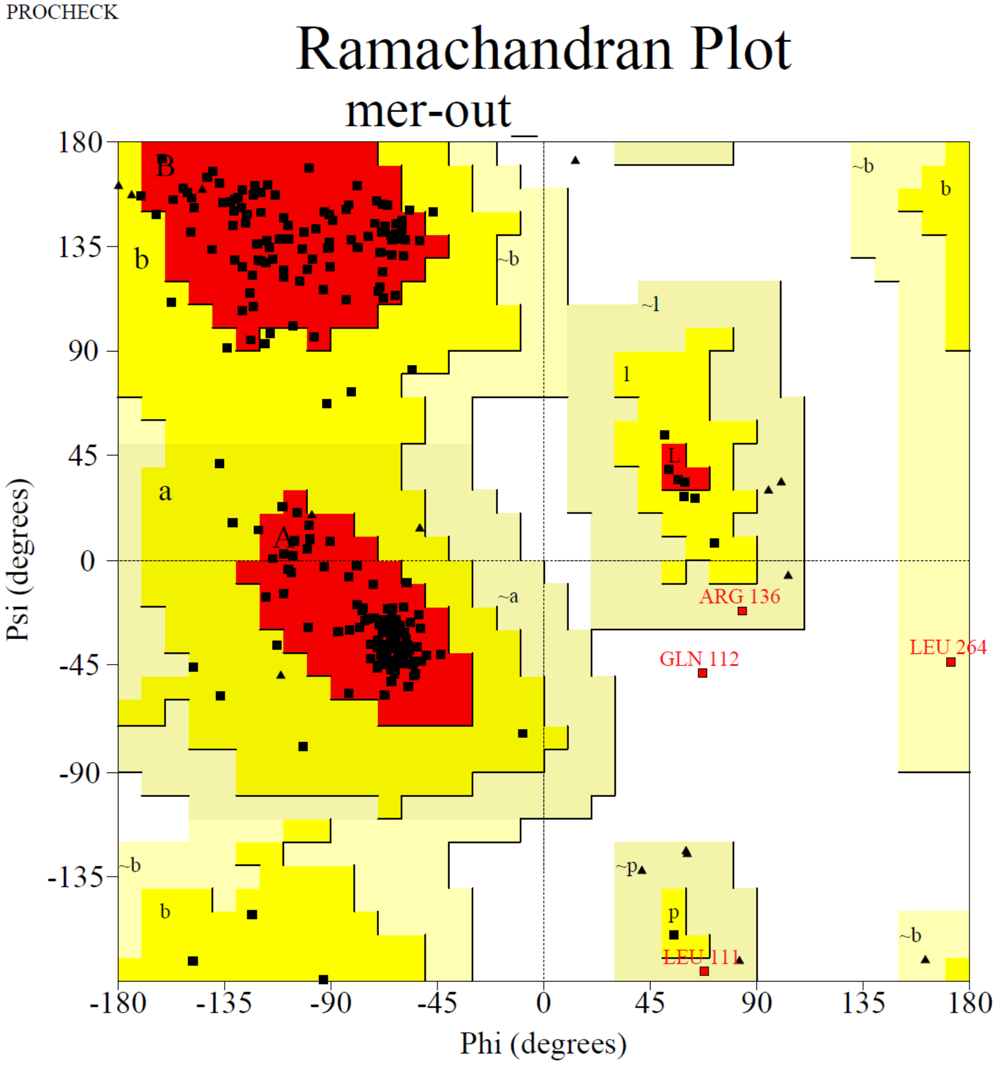

Plot statistics

Residues in most favoured regions [A,B,L]	204	86.8%
Residues in additional allowed regions [a,b,l,p]	27	11.5%
Residues in generously allowed regions [~a,~b,~l,~p]	3	1.3%
Residues in disallowed regions	1	0.4%
	----	------
Number of non-glycine and non-proline residues	235	100.0%
Number of end-residues (excl. Gly and Pro)	2	
Number of glycine residues (shown as triangles)	18	
Number of proline residues	12	

Total number of residues	267	

Based on an analysis of 118 structures of resolution of at least 2.0 Angstroms and R-factor no greater than 20%, a good quality model would be expected to have over 90% in the most favoured regions.

mer-out_

Table S1. TAM DFG-in inhibitors from the databases PubChem and CanSAR. Columns 6 to 10 were built using logical functions to compare the pan-TAM and the Tyro-3-, Axl- or Mer-selective inhibitors. *Pubchem ID* is the compound identifier in Pubchem. *Ligand#* is the ligand numbering. Tyro-3 IC_{50}, Axl IC_{50} and Mer IC_{50}: the ligand IC_{50} values against Tyro-3, Axl and Mer, respectively. Ratio is defined as: ((Tyro-3 IC_{50} * Axl IC_{50})/Mer IC_{50}). *PAN* describes the pan character of the inhibitor: if the inhibitor is a pan-TAM inhibitor, them the value is "TRUE"; if not, the value is "FALSE". *Tyro-3-Selective, Axl-Selective* and *Mer-Selective* all describe the selectivity of the inhibitors: for instance, if the inhibitor is Tyro-3 selective, then the value for the column "Tyro-3 selective" is "TRUE".

Pubchem ID	Ligand #	Tyro-3 IC_{50}	Axl IC_{50}	Mer IC_{50}	Ratio	Pan	Tyro-3 Selective	Axl Selective	Mer Selective
68212759	4	18	4,1	13	0,44	TRUE	FALSE	FALSE	FALSE
5329102	5	49	9	26	0,65	TRUE	FALSE	FALSE	FALSE
71552942	97	70	3990	775	0,47	FALSE	TRUE	FALSE	FALSE
70789756	104	400	5700	4680	0,10	FALSE	TRUE	FALSE	FALSE
57395116	119	246	18400	2340	0,83	FALSE	TRUE	FALSE	FALSE
71552941	91	360	3200	1920	0,31	FALSE	TRUE	FALSE	FALSE
57395117	126	189	23800	970	4,78	FALSE	TRUE	FALSE	FALSE
68212798	33	60	85	1,1	4 214,88	FALSE	FALSE	FALSE	TRUE
68212462	36	160	110	1,2	12222,22	FALSE	FALSE	FALSE	TRUE
72192165	48	280	260	5,2	2692,31	FALSE	FALSE	FALSE	TRUE
73211763	49	100	270	1,7	9342,56	FALSE	FALSE	FALSE	TRUE
72793332	55	370	370	8	2139,06	FALSE	FALSE	FALSE	TRUE
72551073	57	400	400	2,1	36281,18	FALSE	FALSE	FALSE	TRUE
72551074	61	530	480	9	3140,74	FALSE	FALSE	FALSE	TRUE
72793399	62	700	530	6,3	9347,44	FALSE	FALSE	FALSE	TRUE
72792971	63	84	550	1,8	14259,26	FALSE	FALSE	FALSE	TRUE
72793256	64	480	560	4,1	15990,48	FALSE	FALSE	FALSE	TRUE
72547637	67	1200	610	11	6049,59	FALSE	FALSE	FALSE	TRUE
72792560	79	810	930	12	5231,25	FALSE	FALSE	FALSE	TRUE
72793608	80	21900	1100	27	33045,27	FALSE	FALSE	FALSE	TRUE
72551069	86	2200	1700	23	7069,94	FALSE	FALSE	FALSE	TRUE
72547638	87	1700	1900	24	5607,64	FALSE	FALSE	FALSE	TRUE
72793252	90	6700	3000	67	4477,61	FAUX	FALSE	FALSE	TRUE
72793179	92	2930	3460	44	5236,47	FALSE	FALSE	FALSE	TRUE
72793255	98	1900	4300	8,4	115787,98	FALSE	FALSE	FALSE	TRUE
72793611	99	6700	4300	59	8276,36	FALSE	FALSE	FALSE	TRUE
72793610	105	5100	5700	86	3930,50	FALSE	FALSE	FALSE	TRUE
72548104	110	4300	9000	47	17519,24	FALSE	FALSE	FALSE	TRUE
68212509	1	5,9	1,4	0,15	367,11	FALSE	FALSE	FALSE	FALSE
68212505	2	22	3,7	0,76	140,93	FALSE	FALSE	FALSE	FALSE
68212482	3	2,9	4	0,25	185,60	FALSE	FALSE	FALSE	FALSE
68212480	6	14	12	1,8	51,85	FALSE	FALSE	FALSE	FALSE
68212724	7	260	14	18	11,23	FALSE	FALSE	FALSE	FALSE
70681780	8	150	16	3	266,67	FALSE	FALSE	FALSE	FALSE
68212361	9	410	36	25	23,62	FALSE	FALSE	FALSE	FALSE

Table S1. *Cont.*

Pubchem ID	Ligand #	Tyro-3 IC_{50}	Axl IC_{50}	Mer IC_{50}	Ratio	Pan	Tyro-3 Selective	Axl Selective	Mer Selective
53355503	10	48	37	2,9	211,18	FALSE	FALSE	FALSE	FALSE
72792907	11	24	38	0,69	1915,56	FALSE	FALSE	FALSE	FALSE
68212746	12	75	38	8,2	42,39	FALSE	FALSE	FALSE	FALSE
68212733	13	370	38	10	140,60	FALSE	FALSE	FALSE	FALSE
71289225	14	27	42	1	1134,00	FALSE	FALSE	FALSE	FALSE
68212411	15	22	46	0,65	2395,27	FALSE	FALSE	FALSE	FALSE
70692345	16	77	47	14	18,46	FALSE	FALSE	FALSE	FALSE
68212395	17	120	50	56	1,91	FALSE	FALSE	FALSE	FALSE
71289211	18	47	55	1,1	2136,36	FALSE	FALSE	FALSE	FALSE
68212539	19	27	59	3,5	130,04	FALSE	FALSE	FALSE	FALSE
68212791	20	340	59	10	200,60	FALSE	FALSE	FALSE	FALSE
71552696	21	270	61	126	1,04	FALSE	FALSE	FALSE	FALSE
71289190	22	35	62	1,8	669,75	FALSE	FALSE	FALSE	FALSE
72792563	23	21	63	1,7	457,79	FALSE	FALSE	FALSE	FALSE
68212765	24	31	67	0,8	3245,31	FALSE	FALSE	FALSE	FALSE
68212514	25	28	69	1,2	1341,67	FALSE	FALSE	FALSE	FALSE
72792909	26	21	70	0,81	2240,51	FALSE	FALSE	FALSE	FALSE
68212461	27	47	71	1,8	1029,94	FALSE	FALSE	FALSE	FALSE
72792564	28	28	72	0,7	4114,29	FALSE	FALSE	FALSE	FALSE
68212799	29	38	75	1,1	2355,37	FALSE	FALSE	FALSE	FALSE
67471113	30	1400	81	56	36,16	FALSE	FALSE	FALSE	FALSE
68212760	31	2800	83	210	5,27	FALSE	FALSE	FALSE	FALSE
72792908	32	33	84	1,3	1640,24	FALSE	FALSE	FALSE	FALSE
68212757	34	71	100	5,8	211,06	FALSE	FALSE	FALSE	FALSE
68212768	35	35	110	4	240,63	FALSE	FALSE	FALSE	FALSE
68212426	37	68	120	3,2	796,88	FALSE	FALSE	FALSE	FALSE
72792562	38	36	130	1,1	3867,77	FALSE	FALSE	FALSE	FALSE
71289192	39	130	140	18	56,17	FALSE	FALSE	FALSE	FALSE
68212353	40	1500	150	120	15,63	FALSE	FALSE	FALSE	FALSE
72792906	41	94	170	3,4	1382,35	FALSE	FALSE	FALSE	FALSE
72792972	42	58	180	4,5	515,56	FALSE	FALSE	FALSE	FALSE
68212755	43	80	180	13	85,21	FALSE	FALSE	FALSE	FALSE
72793606	44	88	180	2,8	2020,41	FALSE	FALSE	FALSE	FALSE
68212446	45	82	190	50	6,23	FALSE	FALSE	FALSE	FALSE
70681769	46	2100	220	170	15,99	FALSE	FALSE	FALSE	FALSE
72793607	47	110	260	3,9	1880,34	FALSE	FALSE	FALSE	FALSE
72793609	50	940	300	73	52,92	FALSE	FALSE	FALSE	FALSE
68212410	51	45	350	1,1	13016,53	FALSE	FALSE	FALSE	FALSE
68212409	52	430	360	54	53,09	FALSE	FALSE	FALSE	FALSE
68212489	53	450	360	62	42,14	FALSE	FALSE	FALSE	FALSE
72793397	54	240	370	6,7	1978,17	FALSE	FALSE	FALSE	FALSE
68212780	56	13000	380	860	6,68	FALSE	FALSE	FALSE	FALSE
72793111	58	80	430	14	175,51	FALSE	FALSE	FALSE	FALSE
71289195	59	280	450	23	238,19	FALSE	FALSE	FALSE	FALSE
72793605	60	270	460	6,3	3129,25	FALSE	FALSE	FALSE	FALSE

Table S1. *Cont.*

Pubchem ID	Ligand #	Tyro-3 IC$_{50}$	Axl IC$_{50}$	Mer IC$_{50}$	Ratio	Pan	Tyro-3 Selective	Axl Selective	Mer Selective
72793337	65	450	570	14	1308,67	FALSE	FALSE	FALSE	FALSE
72793257	66	540	580	13	1853,25	FALSE	FALSE	FALSE	FALSE
68212763	68	4300	610	400	16,39	FALSE	FALSE	FALSE	FALSE
68212406	69	810	620	47	227,34	FALSE	FALSE	FALSE	FALSE
72793105	70	440	650	19	792,24	FALSE	FALSE	FALSE	FALSE
68212421	71	250	660	140	8,42	FALSE	FALSE	FALSE	FALSE
68212479	72	11000	690	1400	3,87	FALSE	FALSE	FALSE	FALSE
68212750	73	5400	750	530	14,42	FALSE	FALSE	FALSE	FALSE
71289209	74	320	780	18	770,37	FALSE	FALSE	FALSE	FALSE
72547636	75	16200	780	37	9230,09	FALSE	FALSE	FALSE	FALSE
72793400	76	570	790	14	2297,45	FALSE	FALSE	FALSE	FALSE
72792973	77	280	830	18	717,28	FALSE	FALSE	FALSE	FALSE
72793334	78	660	880	15	2581,33	FALSE	FALSE	FALSE	FALSE
72547635	81	30000	1200	150	1600,00	FALSE	FALSE	FALSE	FALSE
72792975	82	390	1280	72	96,30	FALSE	FALSE	FALSE	FALSE
68212498	83	370	1400	74	94,59	FALSE	FALSE	FALSE	FALSE
72792974	84	430	1580	34	587,72	FALSE	FALSE	FALSE	FALSE
72793335	85	830	1700	29	1677,76	FALSE	FALSE	FALSE	FALSE
68212779	88	30000	2400	2400	12,50	FALSE	FALSE	FALSE	FALSE
72792977	89	1340	2790	200	93,47	FALSE	FALSE	FALSE	FALSE
72793047	93	2580	3530	170	315,13	FALSE	FALSE	FALSE	FALSE
72792976	94	1370	3700	160	198,01	FALSE	FALSE	FALSE	FALSE
72793336	95	1500	3800	39	3747,53	FALSE	FALSE	FALSE	FALSE
72793046	96	6850	3920	540	92,09	FALSE	FALSE	FALSE	FALSE
68212528	100	30000	4600	7000	2,82	FALSE	FALSE	FALSE	FALSE
72793466	101	620	4700	37	2128,56	FALSE	FALSE	FALSE	FALSE
68212789	102	730	5000	130	215,98	FALSE	FALSE	FALSE	FALSE
56835089	103	598	5250	2440	0,53	FALSE	FALSE	FALSE	FALSE
72793048	106	1590	6180	50	3930,48	FALSE	FALSE	FALSE	FALSE
71289188	107	290	6500	9,7	20034,01	FALSE	FALSE	FALSE	FALSE
72547868	108	2300	7400	180	525,31	FALSE	FALSE	FALSE	FALSE
72793106	109	370	8040	44	1536,57	FALSE	FALSE	FALSE	FALSE
72793042	111	4910	9890	1130	38,03	FALSE	FALSE	FALSE	FALSE
72793468	112	2000	10700	62	5567,12	FALSE	FALSE	FALSE	FALSE
72793469	113	2000	12600	110	2082,64	FALSE	FALSE	FALSE	FALSE
72793467	114	1600	14000	140	1142,86	FALSE	FALSE	FALSE	FALSE
72793178	115	6140	15000	220	1902,89	FALSE	FALSE	FALSE	FALSE
72793333	116	17900	16600	230	5617,01	FALSE	FALSE	FALSE	FALSE
72793536	117	30000	16600	2400	86,46	FALSE	FALSE	FALSE	FALSE
68212494	118	17000	17000	4500	14,27	FALSE	FALSE	FALSE	FALSE
72793177	120	8940	18500	320	1615,14	FALSE	FALSE	FALSE	FALSE
57396882	121	476	19400	6930	0,19	FALSE	FALSE	FALSE	FALSE
72793109	122	1160	20300	83	3418,20	FALSE	FALSE	FALSE	FALSE
72793043	123	4060	20800	600	234,58	FALSE	FALSE	FALSE	FALSE
72793533	124	4100	21000	620	223,99	FALSE	FALSE	FALSE	FALSE

Table S1. *Cont.*

Pubchem ID	Ligand #	Tyro-3 IC50	Axl IC50	Mer IC50	Ratio	Pan	Tyro-3 Selective	Axl Selective	Mer Selective
70686029	125	75000	22000	2800	210,46	FALSE	FALSE	FALSE	FALSE
68212766	127	720	26000	100	1872,00	FALSE	FALSE	FALSE	FALSE
70696465	128	30000	30000	28000	1,15	FALSE	FALSE	FALSE	FALSE
59604787	130	10000	10000	10000	1,00	FALSE	FALSE	FALSE	FALSE
66553589	131	10000	10000	10000	1,00	FALSE	FALSE	FALSE	FALSE
70697396	132	30000	10000	2200	61,98	FALSE	FALSE	FALSE	FALSE
54915268	133	30000	10000	3000	33,33	FALSE	FALSE	FALSE	FALSE
72793110	134	300	30000	110	743,80	FALSE	FALSE	FALSE	FALSE
72793532	135	19500	30000	1000	585,00	FALSE	FALSE	FALSE	FALSE
72547864	136	30000	30000	120	62500,00	FALSE	FALSE	FALSE	FALSE
72793534	137	30000	30000	170	31141,87	FALSE	FALSE	FALSE	FALSE
72793107	138	30000	30000	380	6232,69	FALSE	FALSE	FALSE	FALSE
72793401	139	30000	30000	590	2585,46	FALSE	FALSE	FALSE	FALSE
70695379	140	30000	30000	2400	156,25	FALSE	FALSE	FALSE	FALSE
72793254	141	30000	30000	2500	144,00	FALSE	FALSE	FALSE	FALSE
72793045	142	30000	30000	2700	123,46	FALSE	FALSE	FALSE	FALSE
72793253	143	30000	30000	5900	25,85	FALSE	FALSE	FALSE	FALSE
70681768	144	30000	30000	9600	9,77	FALSE	FALSE	FALSE	FALSE
72793535	145	30000	30000	9900	9,18	FALSE	FALSE	FALSE	FALSE
72551070	146	30000	30000	12600	5,67	FALSE	FALSE	FALSE	FALSE
72793108	147	30000	30000	16500	3,31	FALSE	FALSE	FALSE	FALSE
72548105	148	30000	30000	30000	1,00	FALSE	FALSE	FALSE	FALSE
72792559	149	30000	30000	30000	1,00	FALSE	FALSE	FALSE	FALSE
72793044	150	30000	30000	30000	1,00	FALSE	FALSE	FALSE	FALSE
72793612	151	30000	30000	30000	1,00	FALSE	FALSE	FALSE	FALSE

Table S2. TAM DFG-out inhibitors from the databases PubChem and CanSAR. *Pubchem ID* is the compound identifier in Pubchem. *Ligand#* is the ligand numbering. Tyro-3 IC_{50}, Axl IC_{50} and Mer IC_{50}: the ligand IC_{50} values against Tyro-3, Axl and Mer, respectively. Ratio is defined as: ((Tyro-3 IC_{50} * Axl IC_{50})/Mer IC_{50}). *PAN* describes the pan character of the inhibitor: if the inhibitor is a pan-TAM inhibitor, them the value is "TRUE"; if not, the value is "FALSE". *Tyro-3-Selective, Axl-Selective* and *Mer-Selective* all describe the selectivity of the inhibitors: for instance, if the inhibitor is Tyro-3 selective, then the value for the column "Tyro-3 selective" is "TRUE".

CID	Ligand 3	Tyro3	AXL	MER	PAN	Selective Tyro3	Selective Axl	Selective Mer
		Kd(nM)	Kd(nM)	Kd(nM)				
71579391	155	900	2100	190	FALSE	FALSE	FALSE	TRUE
71579393	156	2900	170	260	FALSE	FALSE	FALSE	FALSE
71579392	153	200	39	42	FALSE	FALSE	FALSE	FALSE
		IC_{50} (nM)	IC_{50} (nM)	IC_{50} (nM)				
24794418	154	4.3	1.1	14	PAN	FALSE	FALSE	FALSE
44603533	152	28	2	10	PAN	FALSE	FALSE	FALSE
